# Supplementary material for: Genomic Context of SARS-CoV-2 Outbreaks in Farmed Mink in Spain during Pandemic: Unveiling Host Adaptation Mechanisms
Source: Int J Mol Sci. 2024 May 17;25(10):5499. doi: 10.3390/ijms25105499 (PMC11122236; doi:10.3390/ijms25105499)
Supplement: Supplementary file 1 [file ijms-25-05499-s001.zip › ijms-2980834-supplementary.pdf]

Supplementary Material.

Sample ID	Cq control for sequencing	Mean depth	% Coverage	Quality status	Spike sequencing
R.001630/20-091	24.1	989.5	71%	Good	NA
R.001630/20-092	26.2	39.5	15%	NA	No
R.001630/20-093	26.4	1103.5	15%	NA	No
R.001630/20-094	22.3	7250	88%	Good	NA
R.001630/20-096	24.0	7746.8	85%	NA	No
R.001630/20-097	23.7	1271.9	30%	NA	No
R.001630/20-100	28.2	371.6	39%	NA	No
R.001630/20-102	26.7	1236.7	72%	Mediocre	NA
R.001630/20-103	24.8	6166.6	78%	Good	NA
R.001630/20-104	24.6	8703.5	85%	Good	NA
R.001630/20-109	28.2	67.2	22%	NA	No
R.001630/20-115	19.8	8849.0	86%	Mediocre	NA
R.001630/20-117	23.9	6519.8	78%	Good	NA
R.001630/20-124	22.6	5063.4	80%	Good	NA
R.001630/20-125	19.8	9568.6	84%	Good	NA
R.001630/20-126	22.4	4866.6	71%	Good	NA
R.001630/20-127	20.7	4637.3	75%	Good	NA
R.001630/20-128	22.7	423.6	26%	NA	No
R.001630/20-131	22.8	4.3	2%	NA	No
R.001630/20-132	28.4	462.3	37%	NA	No
R.001630/20-133	28.5	11.3	2%	NA	No
R.001630/20-134	26.2	5.2	4%	NA	No
R.001630/20-135	28.4	172.3	13%	NA	No
R.001630/20-137	24.0	3080.3	70%	Good	NA
R.001630/20-139	22.5	8835.0	75%	Good	NA
R.001630/20-141	25.1	4101.1	66%	Good	NA
R.001630/20-142	26.6	1759.3	62%	NA	No
R.001630/20-143	19.9	5310.1	82%	Good	NA
R.001630/20-144	18.2	7470.0	90%	Good	NA
R.001630/20-145	21.3	13317.9	86%	Mediocre	NA
R.001630/20-146	28.5	4362.1	75%	Mediocre	NA
R.001630/20-148	26.0	3230	71%	Mediocre	NA
R.001630/20-150	23.5	8285.3	74%	Good	NA
R.001630/20-151	24.4	8246.9	75%	Good	NA
R.001630/20-152	18.2	7089.8	90%	Good	NA
R.001630/20-154	27.1	2816.4	28%	NA	No
R.001630/20-155	25.6	1757.5	73%	Good	NA
R.001630/20-157	18.3	10022.9	84%	Good	NA
R.001630/20-158	25.4	5282.1	80%	Good	NA
R.001630/20-161	21.3	8358.3	82%	Good	NA

R.001630/20-163	18.1	12905.8	86%	Good	NA
R.001630/20-165	23.5	1337.7	43%	NA	No
R.001630/20-167	21.8	5968	80%	Good	NA
R.001630/20-168	27.0	4070.8	74%	Good	NA
R.001630/20-169	26.9	6282.9	71%	Good	NA
R.001630/20-172	27.2	1157.3	50%	NA	No
R.001630/20-173	18.8	14206.5	91%	Good	NA
R.001630/20-174	27.8	802.4	37%	NA	No
R.001630/20-175	23.6	7396.1	72%	Good	NA
R.001630/20-177	25.6	5369.9	71%	Good	NA
R.001630/20-178	21.8	11843.6	83%	Good	NA
R.001630/20-179	19.2	14381.9	95%	Good	NA
R.001630/20-180	28.5	2029.7	40%	NA	No
R.004000/20-01	26.45	699.94	81%	NA	No
R.004000/20-04	37.02	119.8	22%	NA	No
R.004000/20-08	24.73	282.41	74%	Mediocre	NA
R.004000/20-09	30.33	46.56	24%	NA	Yes
R.004000/20-15	24.03	326.49	77%	Good	NA
R.004000/20-16	25.83	641.96	82%	Mediocre	NA
R.004000/20-17	36.38	50.12	24%	NA	No
R.000128/21-03	16.36	318.18	78%	Good	NA
R.000128/21-19	37.6	516.24	80%	NA	No
R.000776/21-17	21.11	942.2	91%	Good	NA
R.000776/21-18	23.05	516.5	82%	Good	NA
R.000871/21-58	27.22	426.4	72%	Good	NA
R.000871/21-62	22.81	699.1	94%	Good	NA
R.000871/21-68	19.25	1267.7	99%	Good	NA
R.000871/21-69	27.86	418.3	40%	NA	Yes
R.000871/21-82	29.6	203.7	23%	NA	No
R.000871/21-97	23.22	712.3	94%	Good	NA
R.000871/21-99	24.1	87.4	16%	NA	No
R.000871/21-106	25.55	313.5	57%	NA	Yes
R.000871/21-114	21.26	967.2	99%	Good	NA
R.000871/21-116	23.5	740.8	92%	Good	NA
R.000871/21-120	27.66	56.2	28%	NA	No
R.001042/21-32	25.39	268.4	48%	NA	Yes
R.001042/21-78	22.42	706.4	96%	Good	NA
R.001677/21-15	19.81	1442.6	93%	Good	NA
R.001677/21-51	27.62	44	4%	NA	No
R.001677/21-52	23.74	550.2	76%	Good	NA
R.001677/21-67	24.73	500.1	82%	Good	NA
R.220721/3	19.84	6249.6	99%	Good	NA
R.220721/22	20.41	6117.9	99%	Good	NA
R.220721/36	19.94	4443.2	98%	Good	NA
R.220721/39	21.62	6127.3	99%	Good	NA

R.220721/46	19.52	7467.4	99%	Good	NA
R.050821/22	21.62	6788.1	99%	Good	NA
R.050821/58	22.03	1142.5	100%	Good	NA
R.190821/56	24.93	2105.4	92%	Good	NA
R.001738/21-19	22.13	611.5	78%	Mediocre	NA
R.001738/21-21	21.30	866.4	87%	Mediocre	NA
R.001807/21-59	23.06	875.9	70%	Good	NA
R.002026/21-100	25.16	1812.99	90%	Good	NA
R.002026/21-101	25.64	1174.76	75%	Good	NA
R.003152/21-16	25.12	70.74	16.61%	NA	No
R.003152/21-86	27.41	45.05	11.40%	NA	No
R.003152/21-85	27.44	18.45	5.73%	NA	No
R.002083/21-33	27.57	558.29	86.9	Good	NA
R.002366/21-14	27.14	24.35	13.79	NA	No
R.002366/21-27	27.36	206.86	76.29	NA	No
R.002295/21-110	29.53	7.36	3.27	NA	No
R.002295/21-107	25.30	65.54	15.7	NA	No
R.002843/21-09	26.20	69.58	23.57%	NA	No
R.002843/21-12	26.10	13.84	7.02%	NA	No
R.002843/21-14	27.80	135.48	20.15%	NA	No
R.002843/21-20	27.92	13.19	5.57%	NA	No
R.002843/21-23	28.52	13.27	5.90%	NA	No
R.002843/21-41	27.57	17.48	6.43%	NA	No
R.002843/21-71	28.16	40.8	22.23%	NA	No
R.002843/21-75	27.60	17.23	10.10%	NA	No
R.002721/21-61	27.44	8.53	4.59%	NA	No
R.002721/21-106	29.01	65.3	8.92%	NA	No
R.002990/21-01	16.41	2829.9	94%	Mediocre	NA
R.003064/21-01	24.46	87.62	34%	NA	Yes
R.003064/21-33	23.7	99.41	28%	NA	Yes
R.003234/21-26	26.1	124.6	22%	NA	Yes
R.002651/21-32	28.3	66.74	-	-	No
R.002651/21-61	23.9	151.2	35%	NA	Yes
R.002651/21-76	29.22	132.6	22%	NA	Yes

Table S1. Table describing cycle threshold (cq) previous to sequencing, the mean depth of the sequences, the % of coverage and their quality status. NA1: Low amount of RNA in the original sample. No sequencing attempted. NA2: Partial sequence obtained. Lineage cannot be assigned. NA: No apply.

HexaPro variant spike	Mutations	Yield (mg/ml)
**Mink Cluster V**	Δ 69-70 / Y453F / D614G	5.3
**B.1**	D614G	10.1
**B.1 N501T**	N501T / D614G	8.5
**B.1 F486L / N501T**	F486L / N501T / D614G	10.4
**B.1 F486V / N501T**	F486V / N501T / D614G	10.0
**B.1 VTH**	F486V / N501T / D796 H / D614G	14.5
**B.1.177**	A222V / D614G	6.8
**B.1.177 Y453F**	A222V / Y453F / D614G	13.2
**B.1.1.7†**	Δ69-70 / Δ144 / N501Y / A570D / D614G	16.5
**B.1.1.7† E484K**	Δ69-70 / Δ144 / E484K / N501Y / A570D / D614G	14.6

Table S2: Mutations and expression yield of HexaPro variant spikes produced in 293F cells. † Alpha constructs used in the study do not contain the S2 subunit lineage-defining mutation D950N

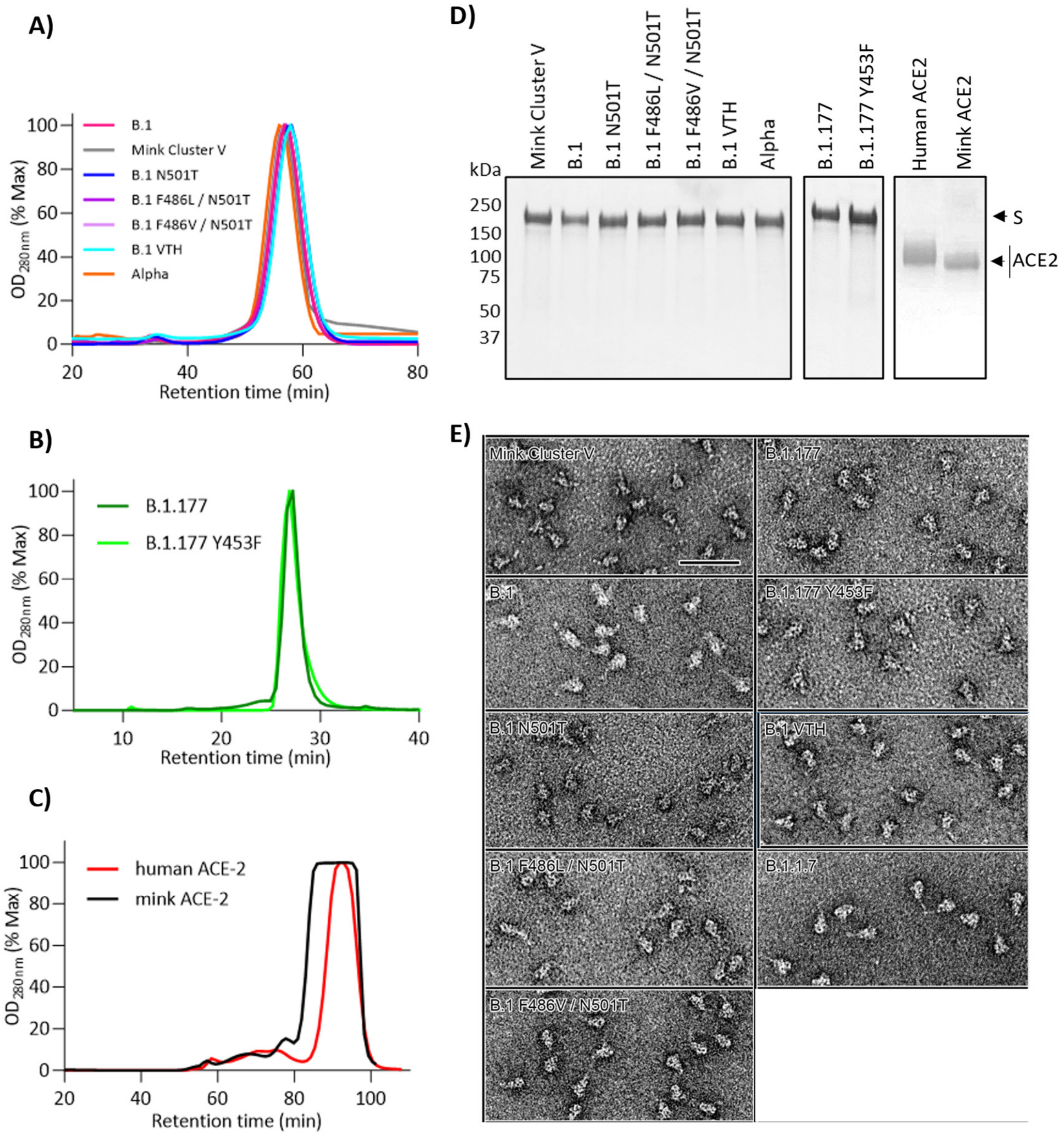

Figure S1. Purity and integrity of HexaPro spikes and ACE2 receptors produced in 293F cells. Gel-filtration traces of the indicated protein preparations run on a Superose 6 pg (A), Superose 6 Increase (B) or Superdex G200 H16/60 (C) columns. D) BlueSafe-stained SDS-PAGE of the major peak (spike trimer or ACE2 monomer) of each chromatogram, run under reducing conditions. Numbers on the left correspond to molecular mass markers (kilodalton, kDa). The position of the uncleaved S and ACE2 bands are indicated on the right. E) Negatively stained (1% uranyl formate) electron micrographs of HexaPro trimers. Electron micrographs of all variant spikes displayed the prefusion conformation of SARS-CoV-2 S protein. Images were recorded on a Gatan ES1000W charge-coupled-device (CCD) camera in a JEOL JEM-1011 microscope operated at 100 kV. Scale bar: 50nm.

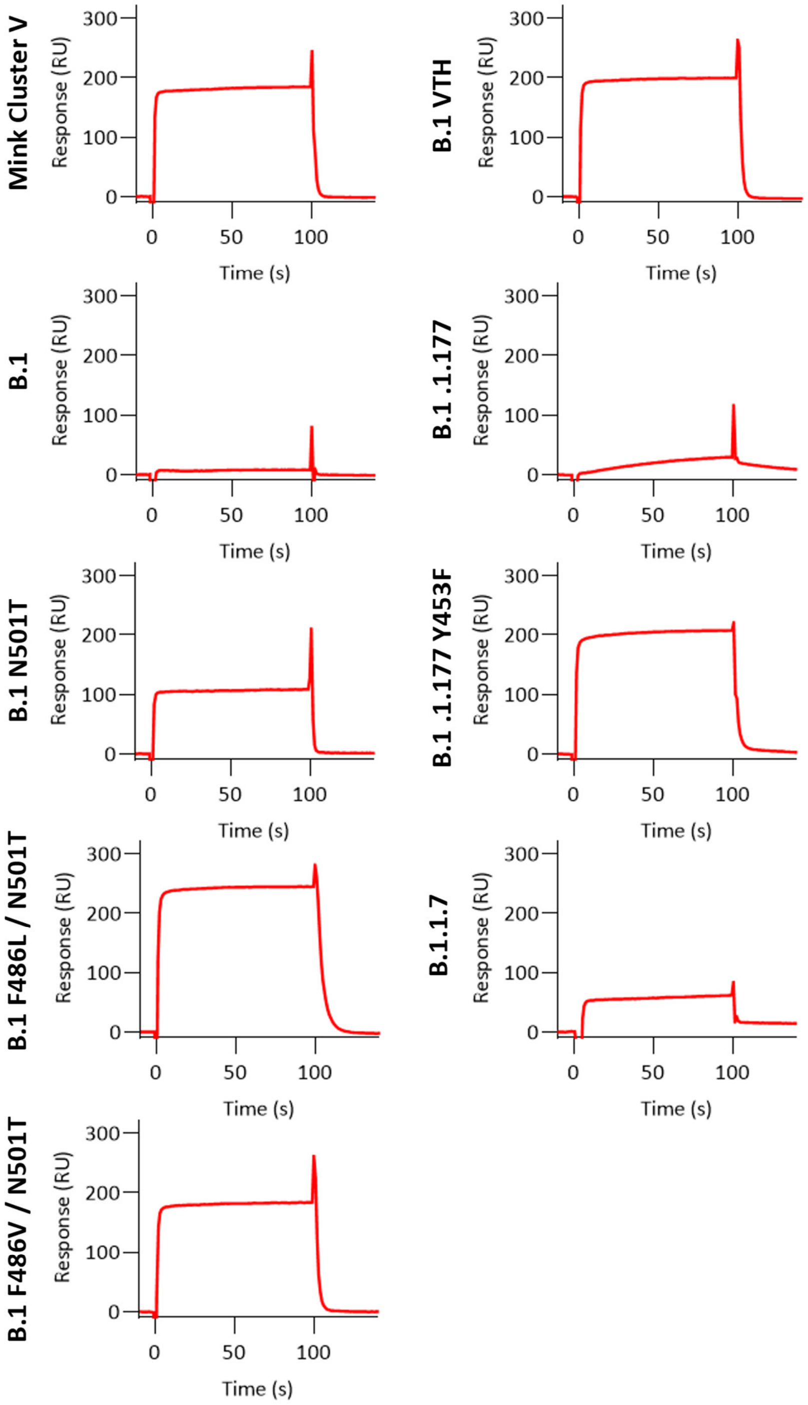

Figure S2. Binding of the indicated purified spikes to the mink ACE2 evaluated by surface plasmon resonance. A single trace for one replicate at the mkACE2 concentration of 50mM is shown. RU, response untis.

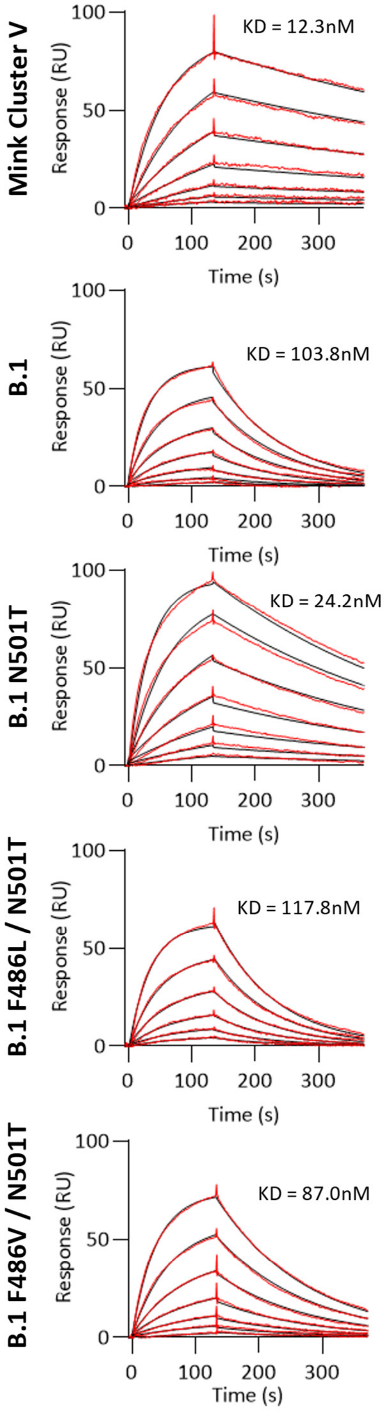

Figure S3. Affinity of the indicated purified spikes to the hACE2 measured by surface plasmon resonance. Sensorgrams of seven different hACE2 concentrations are shown for one run replicate in each panel. Red lines indicate the raw data and black lines the fit to the "Langmuir 1:1" kinetic model. For each spike the calculated affinity constant (KD) is shown. RU, response untis.
